# Developing a national health research system: participatory approaches to legislative, institutional and networking dimensions in Zambia

**DOI:** 10.1186/1478-4505-10-17

**Published:** 2012-06-06

**Authors:** Pascalina Chanda-Kapata, Sandy Campbell, Christina Zarowsky

**Affiliations:** 1 Directorate of Public Health and Research, Ministry of Health, Lusaka, Zambia; 2 Independent Consultant, Berkeley, CA, USA; 3 University of Western Cape, Cape Town, South Africa

**Keywords:** Health, Research, Systems

## Abstract

For many sub-Saharan African countries, a National Health Research System (NHRS) exists more in theory than in reality, with the health system itself receiving the majority of investments. However, this lack of attention to NHRS development can, in fact, frustrate health systems in achieving their desired goals. In this case study, we discuss the ongoing development of Zambia’s NHRS. We reflect on our experience in the ongoing consultative development of Zambia’s NHRS and offer this reflection and process documentation to those engaged in similar initiatives in other settings. We argue that three streams of concurrent activity are critical in developing an NHRS in a resource-constrained setting: developing a legislative framework to determine and define the system’s boundaries and the roles all actors will play within it; creating or strengthening an institution capable of providing coordination, management and guidance to the system; and focusing on networking among institutions and individuals to harmonize, unify and strengthen the overall capacities of the research community.

## Introduction

In the 1990s, the national health research system (NHRS) emerged as a key concept in understanding the systems dynamics of health research at the national level. Beginning with 1990’s Commission on Health Research for Development, the concept of the NHRS has opened windows into the regulatory, governance and institutional environment framing research in low- and middle-income countries [1,2]. Broadly defined here as a system designed to coordinate health research processes, findings, and structures, with the overarching goal of enhancing the health system’s ability to perform its core functions, the NHRS is a critical yet underdeveloped concept^a^.

For many sub-Saharan African countries, an NHRS exists more in theory than in reality, with the health system itself receiving the majority of investments. However, this lack of attention to NHRS development can, in fact, frustrate health systems in achieving their desired goals. A weak NHRS can impede the coordination and harmonization of researchers, institutions, and knowledge; block core stakeholders from participating in research processes; lead to the unethical conduct of research; and further reinforce the separate worlds of researchers and policy-makers, with the latter neither demanding nor accessing research or the wider knowledge base [2-5].

Yet for such an important system, the scientific literature offers little insight, and next-to-no guidance. For a low- and middle-income country seeking to strengthen its NHRS, what are some concrete and tested steps to pursue?

In this case study, we discuss the ongoing development of Zambia’s NHRS. We reflect on our experience in the ongoing consultative development of Zambia’s NHRS and offer this reflection and process documentation to those engaged in similar initiatives in other settings. We argue that three streams of concurrent activity are critical in developing an NHRS in a resource-constrained setting: developing a legislative framework to determine and define the system’s boundaries and the roles all actors will play within it; creating or strengthening an institution capable of providing coordination, management and guidance to the system; and focusing on networking among institutions and individuals to harmonize, unify and strengthen the overall capacities of the research community.

### The national health research system in Zambia

In the literature, an NHRS is often seen through its four principle functions:

· Governance and leadership. A strong NHRS is typically defined in national legislation and led by an institution capable of articulating and enforcing the system’s vision and mission. This institution coordinates the people, institutions and activities within the system through four primary tools: priority setting; establishing and enforcing ethical standards; monitoring and evaluation of the NHRS; and, deepening a culture of evidence-based decision-making [6-8]. Currently, the Ministry of Health is responsible for governance and leadership.

· Financing. A well-functioning NHRS has the ability to source research funding, and disburse those funds equitably, accountably and in line with its overarching vision. Importantly, it can monitor government expenditures for health research, and expenditures from external funding partners [6]. The government funding for health research in Zambia is through the central allocation to the health sector, but there is also external funded research through public and private research organizations and consortiums.

· Strengthening capacity. This occurs at the individual, institutional and systems level, and includes programmes for building capacity in research methods, knowledge translation, and policy-maker abilities to access, assess, adapt and apply research. This can also include improving the physical infrastructure for research, along with intrinsic and extrinsic improvements in career structures to reverse the well-documented brain drain of researchers [7].

· Knowledge generation, translation and utilization. Flowing from the above three functions, the strength of an NHRS can ultimately be seen in the quality of the research its stakeholders produce; in the creation of integrated partnerships of researchers and research-users; in the routine demand of research from policy-makers; in the system-wide abilities to synthesize research (particularly in adapting research from other contexts) and to disseminate it; and in the eventual implementation of evidence-informed policy as well as the creation of more policy-informed research.

In Zambia, as in most of its neighbours, there is widespread acknowledgement that the existing NHRS does not effectively execute any of the above four functions. This recognition has prompted the research community and the government to develop a range of approaches to address these shortcomings. While many of these activities began with 1999’s First National Health Research Agenda [9], it is only in recent years that Zambia has taken strong steps in establishing a base for the robust development of its NHRS. A new National Health Research Bill outlines where health research fits within the government, defining roles and duties; a newly developed institution – the National Health Research Authority of Zambia – will take leadership of health research in the country; and the research community has focused its attention on networking to increase collaboration among existing health researchers and research institutions, further cementing a spirit of harmonization among all domestic and foreign actors involved in health research in Zambia. Critical to each of these streams are multi-disciplinary, multi-stakeholder approaches, with consultation and deliberation a routine and inclusive event shaping the flow of each stream.

### The legislative stream

Legislation is crucial to the definition of an NHRS, providing clarity and consensus on roles and responsibilities. Legislation mandates who has oversight of health research – which ministry, for instance, or which council – thus specifying ownership and providing accountability. As in many other sub-Saharan African countries, health research in Zambia was once the domain of the Ministry of Science, Technology and Vocational Training, with instrumental links to the National Science and Technology Council (NSTC) and the Ministry of Health (MOH). However, the ambiguity of roles and responsibilities contributed directly to the lack of strong leadership, sometimes leading to the duplication of efforts.

While legislation appears to be a first step in the transparent, deliberative approach to developing an NHRS, in fact this represents the end of one long cycle and the beginning of another. Legislation is the culmination of months or years of reflection: in Zambia, it took over three years for the MOH to explicitly articulate the need for a comprehensive legal framework for the institutionalization and administration of health research, resulting in 2008’s National Health Research Policy[10]. This was given formal Cabinet approval in 2010 – a major milestone as it provides strategic direction for the promotion, conduct, prioritization, financing and institutionalization of health research. Critically, the policy recognizes the inability of current organizations – due to human, organizational and financial constraints – to pursue those strategic directions and thus calls for the creation of a national health research coordinating body to achieve its objectives of research coordination and harmonization.

### Ethics in Health Research

In 2007, the MOH began addressiang the outstanding ethical issues surrounding the conduct of health research in Zambia. At this point, it was realized that most of the research was being conducted in the country without ethical approval – directly compromising the protection of human participants. Of particular concern was the unregulated export of biological materials abroad (e.g. blood samples), including the indiscriminate collection of samples for future unknown studies. Additionally, there were no community research advisory boards (CRABS) to provide some guidance to researchers at the community level.

The first step in addressing this problem was the issuance of a circular to all relevant health research institutions – from those involved in health research to those in service delivery – that all health research would need the Ministry of Health clearance before initiation. This would create a screening process for all research activities to ensure their compliance with ethical standards for studies involving human participants. The implementation of this circular, however, revealed many of the shortcomings in the NHRS: there was no standardized way to deal with researchers not following the internationally accepted ethical standards, and inadequate capacity to monitor actual research conducted in the field. In the absence of the legal framework for dealing with research misconduct, the MOH considered halting research involving human beings. In practical application, only ongoing studies were allowed to go on, while new research activities were temporarily banned. While not totally enforced, this moratorium was not lifted until February 2011, when Zambia’s draft National Health Research Bill was finally developed. As of writing, this Bill awaits enactment into law by Parliament.

In the meantime, the MOH created the National Health Research Ethics Committee (NHREC) to assist in the development, monitoring and enforcement of ethical standards. The NHREC is a multi-disciplinary body, funded by MOH but operating independently in executing its ethics mandate. The formation of NHREC has provided a system for the regulation, capacity building and coordination of activities of various institutional review boards. NHREC is also responsible for the regulation of biological material transfer for research purposes. Once the research bill is enacted, NHREC will be institutionalized within the National Health Research Authority of Zambia.

### The institutional stream

A national-level institution, with the mandate to coordinate all research activities, mobilize funds to support priority research, disseminate key findings, and advocate at a policy level, had been under discussion in Zambia since 1999. Until 2008, however, little came out of the many deliberations, with agreements and ideas not implemented principally due to lack of resources.

Recognizing some of the fundamental challenges in implementing policy measures in the absence of a formal regulatory framework, in 2008 the MOH established an internal unit responsible for coordinating health research in Zambia. To date, it has been assisted in its mandate by the ad-hoc, multi-disciplinary National Health Research Advisory Committee (NHRAC), which advises the MOH on all issues related to research, and comprised the bulk of the membership for a Technical Working Group (TWG) charged with discussing the creation of a national-level institution to govern health research in the country.

In 2009, with technical support from the Canadian Coalition for Global Health Research (CCGHR) and financial support from Canada’s International Development Research Centre (IDRC), a two-year participatory process began – involving stakeholders from across the health sector – to develop an operational strategy for what is now known as the National Health Research Authority of Zambia (NHRAZ)^b^. Among other things, the participatory process discussing the NHRAZ sought to determine its exact nature, the range of services it could offer, whom it might serve and how, the funding arrangements it would follow, its governance structure, legal status and sustainability. As in other African countries^c^, overseeing and leading this process was an inclusive, multi-stakeholder Technical Working Group (TWG) charged with discussing the existing state of research and how the NHRAZ could work to build the system’s capacity over the long term.

To provide transparency and a formal structure to the TWG’s brainstorming and deliberations, an external facilitator led the TWG using Visualization in Participatory Programmes (VIPP) techniques [11,12]. Here, cards, diagrams and photographs were used to express main ideas, with the “less talkative” participants encouraged to express themselves, and the group able to arrive at a genuine consensus on highly complex issues^d^. With the ultimate goal of creating a Strategic Plan and Resource Mobilization strategy for the NHRAZ, TWG members worked through the following sets of activities:

a. Situation analysis. Following group discussions about Zambia’s NHRS – its abilities, needs and challenges – the TWG commissioned a consultant to study the wider landscape, including an investigation of prior attempts to form an overarching body; the range of institutions and organizations involved in health research; a “white pages” of individuals involved in health research; and an analysis of how health research has or has not influenced health policy in the country.

b. Field visits. This element of the process saw sets of two TWG members (not from the same institution) visit domestic organizations to gather structured information related to their position within the wider health research community, their successes, challenges, and their leaders’ opinions on NHRAZ’s possible niche. How best could NHRAZ assist the strengthening of Zambia’s NHRS?

c. Comparator visits. Similar to the idea behind field visits, at this stage members of the TWG visited leading institutions in other African countries to learn from their challenges and vantage-point. These visits provided critical snapshots on some of the key issues other institutions have grappled with in their formation and operation.

d. Expert Witnesses. Identified by the TWG as having information critical to the formation of the NHRAZ, expert witnesses attended TWG meetings for the sole purpose of addressing the questions from the TWG panel – as a witness might in a courtroom. Questions revolved around issues related to an organization’s initial formation, funding arrangements for research institutions, priority setting, regulatory and coordinating function, and so on. This proved a highly efficient means of extracting substantial and salient knowledge or opinion from key individuals – subtracting the need for lengthy presentations and speeches that might not address core issues of need for the TWG.

Following this lengthy deliberative process, the TWG developed recommendations for the administrative and legislative needs central to NHRAZ’s creation.

### Legal Framework for the NHRAZ

Concurrent with these activities, the Ministry of Health developed the National Health Research Bill to provide a legal framework for the development, coordination, financing, dissemination and regulation of health research in Zambia. Some members of the NHRAC/TWG were co-opted to work with the technical drafting committee to provide recommendations derived from the NHRAZ report. The National Health Research Bill, once enacted will provide, among other things, for the formation of the NHRAZ, including its powers and how it will be financed. In this process, again we see the role of high-level political involvement and commitment to address gaps in the health research system, without which, the process may well have faced insurmountable obstacles. Within six months of approval of the National Health Research Policy, the National Health Research Bill underwent formal processes of enactment to govern the health research system.

#### Administrative Framework for NHRAZ

The NHRAZ’s administrative framework provides for the human-resource requirements and functioning of the NHRAZ. Various administrative structures were considered, but it was finally agreed that the proposed administrative structure would mimic existing statutory boards in the country (seeing that the NHRAZ would be funded by government). The administrative framework provides for the number and composition of the board of the NHRAZ and allows for employment of relevant personnel for NHRAZ to carry out its coordination, monitoring, priority setting, resource mobilization and other roles.

As of writing (November 2011), the NHRAZ will, upon its formal establishment, develop a framework for how it will:

· Oversee the health-research agenda, from leading priority-setting processes to providing ethical approval for research. This would also include strong enforcement mechanisms to ensure research and researchers follow these protocols.

· Monitor, harmonize, coordinate health research and the health research community

· Actively work to strengthen the capacities of domestic researchers, from convening regular training courses (e.g. on knowledge management, knowledge translation) to sponsoring promising students for higher education

· Advocate for evidence-informed decision-making and policy formulation

· Foster the dissemination of research findings

· Develop effective resource mobilization capacities.

### The networking stream

Many institutions now recognize that they cannot possibly perform all tasks core to their mandate, and that significant duplication could occur were they to ignore avenues for collaboration with like-minded institutions. Networking has been a critical component in the success of the lead health-research funding and coordinating institutions in both Canada and the United States – respectively the Canadian Institutes for Health Research and the National Institutes for Health – with both acting, in effect, as umbrella organizations providing guidance and cohesion to their many networked members and grantees.

Networking provides proven opportunities for researchers to share their knowledge on the conduct, regulation, coordination and financing of health research – and of course on their findings, methodologies and syntheses [13,14]. Networks can be instrumental in the diffusion of innovations, from better clinical practice to the development of new methodologies (e.g. policy briefs and dialogues) to the promotion and achievement of internationally-mandated standards. Through networking, knowledge can be adapted from external sources to complement and build upon the domestic – with particular ramifications for methodology development, capacity strengthening and funding.

In Zambia, the MOH-appointed NHRAC is in itself a network of individuals from across multiple disciplines who work together to advance solutions in health research. The Zambia Forum for Health Research (ZAMFOHR), a non-governmental organization (NGO) launched in 2005, is another attempt at achieving a “more than the sum of the parts” efficiency. ZAMFOHR has had particular value in bringing researchers, research-users and research and health-equity institutions together to engage on research issues with government. Its networking efforts of note include the multi-stakeholder development of policy briefs and dialogues on mental health and reproductive health; and the current development of a Rapid Response Service designed to assist research-users by pairing them with relevant researchers able to offer tailored and highly responsive evidence summaries.

In some ways, this spirit of networking is still in its infancy in Zambia. However, the processes behind the creation of the NHRAZ highlighted the great utility of making these instrumental connections between disparate yet aligned entities, and as such the spirit of networking has been incorporated into NHRAZ as a core foundation.

### Learning from the experience of other countries

The Zambian process described here benefitted enormously from the experience of other countries – particularly Malawi, Kenya and South Africa. This comparative learning process highlighted common and distinctive challenges and opportunities across the different contexts. While all comparator countries – and institutions within them – addressed the three streams discussed in this paper, the relative roles of central oversight, networking, coordination, and decentralization vary.

The timing of the Zambian process was concurrent with like processes in Kenya and Malawi. Both countries were also assessing the state of their national health research systems, and working (with the support of DFID, Wellcome Trust and IDRC) to develop a five-year plan to strengthen individual, institutional, coordination, networking and regulatory capacities. In Malawi, investigators identified an absolute shortage of researchers in nearly all fields, but with existing though often weak structures and mechanisms for ethical review and coordination involving a broad range of key stakeholders. To address this, the investigators opted to work through an existing government body, the National Research Council of Malawi, to promote both increased research production and training, and strengthened coordination. The initial five-year work would focus on strengthening the coordinating body and competitive funding mechanisms, and funding a broad range of research and training.

In Kenya, where the research capacity and productivity is much higher than in Malawi and Zambia, investigators described an NHRS as fragmented, overly competitive and duplicative, lacking health systems-related research or skills, having weak governance and ethical oversight, and marked by official bodies broadly seen as cumbersome and unable to offer incentives for collaboration and innovation. The Kenyan investigators therefore decided to create a new institution which would be arms-length from existing research institutions and from government, but which would work closely with both. The initial five years would focus on building a strong, transparent and collegial institution (the Consortium for National Health Research) providing catalytic funding for interdisciplinary research and training, and strengthening the ethical oversight and regulatory capacity of government.

In South Africa, universities and research centres are both more numerous and stronger than elsewhere in the region. Its NHRS is marked by both competition and collaboration among research institutions and between researchers and government; long experience with multi-stakeholder processes to develop at least some loosely shared research priorities, a strong and almost entirely government-funded National Research Foundation which plays a key role in promoting and funding university-based research, and Provincial and some District Health Research Committees which must approve all research involving public facilities.

## Conclusions

The development of institutional, legal and networking frameworks for health research in Zambia represent major milestones. While the actual implementation of these frameworks remains very much a work in progress, the potential to strengthen Zambia’s NHRS is real. Key lessons and conclusions from the strengthening of Zambia’s NHRS include:

1. The time requirements for this work are significant. A great deal of time is required to understand the status quo, the prevailing context of the health research community, and to define the overarching objectives of an entity like the NHRAZ. In doing so, the process requires the time and expertise of individuals from various backgrounds. Convening the relevant quorum is a challenge as it also involves people who have other engagements in their own right.

2. Given the systemic and overlapping nature of many health challenges, the need for inter-sectoral collaboration must not be underestimated. If not addressed, it could lead to serious gaps in the process which would impact negatively on the outcome by either delaying or terminating the whole process. Such collaboration goes beyond inter-ministerial dynamics to include institutional, individual and inter-country exchanges. This adds value and provides very important information which may easily be overlooked.

3. The process requires adequate financial investments to meet the costs of meetings, travel, communication, consultancies and accommodation or conferencing facilities.

4. Regular updates to the research community are important to facilitate cooperation and also to allay unnecessary opposition to the process. A consultative forum was a valuable source of information from people involved in health research in the country.

5. The steps taken by the Ministry of Health to protect the national interest, safeguard human participants and ensure accountability is important as an NHRS is, like many LIC systems, easily subject to the influences of groups with their own individual or institutional agendas.

6. The VIPP method behind the formation of the NHRAZ was instrumental in creating a forum for dialogue, knowledge and power exchange dynamics, and thus created a sound learning base to realize the objectives of the consultative TWG. This is important because it is easy to have the process unintentionally derailed by individual and institutional attributes [15]. But through the VIPP method, there was a balance of power among the people in the TWG.

The three streams discussed here have begun to converge and thus to strengthen the development of Zambia’s NHRS. The formulation of legal and policy frameworks to govern and guide the health-research community are landmark achievements, and the coming establishment of the NHRAZ will also keep Zambia at the vanguard of resource-constrainted countries designing new architecture for their health research systems. Much certainly remains to be done, but the ongoing developments have already contributed to a vastly improved health-research environment, and will without a doubt bear a strong influence on the future quality, quantity and utilization of health research in the country.

## Endnotes

^a^Definition adapted from Kirigia and Wambebe (2006). A health system’s functions are here understood in accord with the WHO’s six building blocks – information, governance, service delivery, human resources, medicines & technologies, and financing. See Everybody’s Business (2007) for more

^b^Throughout the process, this institution was given many different names, including the National Health Research Agency, the National Health Research Board and the National Health Research Council. The ultimate selection of “Authority” was taken to elevate this entity beyond the usual powers of a Council or Commission.

^c^We make specific reference here to Kenya and Malawi, who under the Health Research Capacity Strengthening Initiative of IDRC, Wellcome Trust and DFID created like-minded institutions using similar participatory processes.

^d^For more on VIPP, see UNICEF 1993 and McKee et al 2009.

## Competing interests

The author(s) declare that they have no competing interests'.

## Authors’ contributions

PCK, SC and CZ contributed to the conceptualization, writing and finalising of the manuscript. All authors read and approved the final manuscript.

## References

[B1] Hanney SR Block MAG Why national health research systems matter HealthResearch Policy and Systems 2008 6 1 doi:10.1186/1478-4505-6-1 10.1186/1478-4505-6-1 PMC2263038 18190714

[B2] Sadana R D’Souza C Why do case studies on national health research systems matter? Identifying common challenges in low- and middle-income countries Social Science & Medicine 2006 62 2072 2078 10.1016/j.socscimed.2005.08.022 16165261

[B3] Kirigia JM Wambebe C Status of national health research systems in ten countries of the WHO African Region BMC Health Services Research 2006 6 135 10.1186/1472-6963-6-135 17052326 PMC1622748

[B4] Hanney SR González Block M Building health research systems to achieve betterhealth Health Research Policy and Systems 2006 4 10 10.1186/1478-4505-4-10 17087830 PMC1636053

[B5] Palmer A Anya SE Bloch P The political undertones of building national health research systems – reflections from The Gambia Health Research Policy and Systems 2009 7 13 10.1186/1478-4505-7-13 19476660 PMC2693112

[B6] Ijsselmuiden C Matlin S Why Health Research? Global Forum for Health Research http://Whqlibdoc.who.int/publications/2006/2940286477_eng.pdf

[B7] Pang T Sadana R Hanney S Bhutta ZA Hyder AA Simon J Knowledge for better health – a conceptual framework and foundation for health research systems The Bulletin of the World Health Organization 2003 81 3 815 820 PMC2572351 14758408

[B8] Sadana R Pang T Current approaches to national health research systems analysis: a brief overview of the WHO health research system analysis intiative Ciencia & Saude Coletiva 2004 9 2 351 362 10.1590/S1413-81232004000200012

[B9] Biemba G Zambia National Health Research Agenda: National Health Research Priorities and Recommendations for Action 1999-2001” 2002 Government of the Republic of Zambia: Central Board of Health

[B10] Ministry of Health. National Health Research Policy Government of the Republic of Zambia 2010 Zambia: Lusaka

[B11] UNICEF Visualisation in Participatory Programmes 1993 Available at: http://portals.wi.wur.nl/files/docs/ppme/VIPP_Unicef.pdf

[B12] McKee N Visualisation in Participatory Programmes (VIPP): Taking stock of its diffusion and impact. Journal of Communication for Development and Social Change 2009 Creskill NJ: Hampton Press

[B13] Mendizabal E Understanding networks: the form and function of research policy networks 2006 ODI Working Paper 271. Available at www.odi.org.uk/resources/download/133.pdf. Accessed June 30, 2009

[B14] Merrill J Findings from an organizational network analysis to support local public health management Journal of Urban Health: Bulletin of the New York Academy of Medicine 2008 2008 85 4 10.1007/s11524-008-9277-8 PMC2443256 18481183

[B15] Smith MK “Bruce W. Tuckman – Forming, storming, norming and performing in groups”. The Encyclopaedia of Informal Education 2005 www.infed.org/thinkers/tuckman.htm. Accessed March 25, 2008

